# Does Samaritan, a Digital Support Platform, Help Improve Social Determinants of Health for Mental Health Offenders in Jacksonville, Florida?

**DOI:** 10.7759/cureus.52915

**Published:** 2024-01-25

**Authors:** Rishubh H Shah, Dionne Blake, Brian Celso, Colleen Bell, Ana Turner

**Affiliations:** 1 Psychiatry, University of Florida College of Medicine, Gainesville, USA; 2 Surgery, University of Florida College of Medicine – Jacksonville, Jacksonville, USA; 3 Psychiatry, HCA Florida Orange Park Hospital, Jacksonville, USA; 4 Psychiatry, University of Florida College of Medicine – Jacksonville, Jacksonville, USA; 5 Psychiatry, University of Florida, Gainesville, USA

**Keywords:** public psychiatry, jail diversion, homelessness, mental health services, social determinants of health (sdoh)

## Abstract

Background

The objective of this study is to evaluate if access to Samaritan, a digital support platform, improves the social determinants of health (SDOH) needs for patients enrolled in a jail diversion program in Jacksonville, FL.

Methodology

A total of 59 patients who were enrolled in a jail diversion program for homeless mentally ill misdemeanor offenders in Jacksonville, FL, participated in the study. Of the 59 patients, 47 individuals consented to participate in Samaritan while 12 declined participation. Demographics and the Health Leads Social Needs Screening Tool scores from the electronic health record were compared between groups along with average financial support from Samaritan. These non-normally distributed variables were compared using Wilcoxon rank-sum tests.

Results

The majority of study participants were male (92%, n = 43). The average age of study participants was 42 years. The average income from donors on the platform over three months for those who opted in was $48.80 (SD = 53.75). Among the individual Health Leads Social Needs Screening Tool questions, intact *Housing *was statistically significant (Z = -2.002, p = 0.045), suggesting access to a digital technology such as Samaritan might help improve SDOH needs.

Conclusions

Access to digital technologies, such as Samaritan, might help offenders with mental illness adjust to the many challenges they face upon reentry into the community. As such, these devices may represent one means for improving SDOH needs for disadvantaged mental health patients.

## Introduction

Interactions between the criminal justice system and mental health offenders (offenders suffering from severe mental illness) can be a dogged challenge. Research indicates that the probability of being arrested is 67% greater in people demonstrating signs of mental illness compared to those without. Moreover, the criminal justice system fails to serve as an effective gateway to mental health services [1]. However, there are programs, such as the Mental Health Offender Program (MHOP) with the Fourth Judicial Circuit and Sulzbacher in Jacksonville, FL, that provide a treatment-based court diversion program with the goal of enhancing mental healthcare and social services for individuals with severe mental illness. MHOP works with the criminal justice system to divert non-violent offenders with severe mental illness from incarceration, redirecting these individuals to the necessary medical care they require.

Over a three-month period (March to May 2023), MHOP worked with the Samaritan platform to improve patient outcomes and foster patient autonomy across each offender’s rehabilitation journey. Samaritan is a digital support platform that allows donors to assist frontline care workers with patient needs [2]. Once the app is downloaded, app owners can learn about participants, along with their needs, and choose to send motivational messages or a monetary contribution toward their goal. By providing instant access to verbal feedback, we hope individuals can regain their sense of self and continue to work toward their goals. Within the Samaritan platform, care workers and case managers can request financial support from donors to address immediate needs tied to the social determinants of health (SDOH). Patients can also complete tasks and goals, such as attending group sessions or having a negative urine drug screen, to earn monetary rewards. These rewards provide patients with discretion over their own funds. A patient’s sense of autonomy and control over the day-to-day of life have been shown to improve healthcare outcomes and patient satisfaction [3]. By offering patients the ability to interact with and receive support from individuals they might not otherwise encounter, this platform hopes to empower them to feel like valued members of the community.

The World Health Organization defines social determinants of health as “the conditions in which people are born, grow, work, live and age” [4]. SDOH also include a wider set of forces and systems that shape conditions of daily life including economic policies and systems, development agendas, social norms, and political systems. Factors cited as having a considerable impact on health inequity include access to nutritious food, neighborhood safety, distance to public transportation, and adequate housing [5]. The purpose of this study is to assess whether accessing Samaritan improves SDOH for MHOP patients utilizing data captured within the Samaritan app as well as the Health Leads Social Need Screening Tool, a validated SDOH screening tool [6]. These tools captured the data points of interest including sociodemographic information, exposure to violence, transportation challenges, financial resource strain, utility needs, housing instability, and food insecurity. While the financial supplementation provided through the Samaritan app is an obvious benefit, this study aims to investigate whether the app can improve SDOH beyond monetary support.

## Materials and methods

Inclusion criteria for this study included any patient admitted to the MHOP team from January 2021 through December 2023, who must be over age 18, and any gender. Criteria for selection into MHOP include having a diagnosis of severe mental illness (schizophrenia, schizoaffective disorder, bipolar disorder, or severe major depressive disorder), experiencing homelessness, being arrested four or more times since 2017, having no sexual offender classification, having no open felony cases, and not currently being on felony probation or parole.

The Samaritan app gathers and stores information about each participant’s SDOH quality measurements. As described by the National Committee for Quality Assurance, these measures include five domains, namely, economic stability, neighborhood/built environment, health and healthcare, education, and social/community context [7]. These domains are also found in the Electronic Medical Record utilized by Sulzbacher. In addition to this information, we also utilized the Health Leads Social Needs Screening Tool.

The Health Leads Social Needs Screening Tool is a validated SDOH screening tool that measures strains on financial resources, utilities, childcare, living arrangements, food access, behavioral health, mental health, need for health assistance, literacy, and social connections [6]. Furthermore, this tool also captures sociodemographic information, exposure to violence, and transportation challenges. We compared Health Leads Social Needs Screening Tool Measures for a 12-month period for MHOP clients who opted to participate in the Samaritan platform to similar MHOP patients who opted not to participate. We also compared different average monthly incomes across participants with their SDOH quality measures to study possible correlations with that metric. Patient demographic data were reported as n (%). Non-normally distributed variables were compared using Wilcoxon rank-sum tests and reported as medians. The level of significance was set at 0.05. All analyses were done in SPSS Version 29.0.0.0 (IBM Corp., Armonk, NY, USA).

## Results

Based on inclusion criteria, data from 59 individuals were utilized for this study, i.e., those who consented to participate in the Samaritan digital support platform (n = 47) and those who declined (n = 12). Basic descriptive statistics were generated for each group. The majority of study participants were male (92%, n = 43). The average age of study participants was 42 years. The only significant difference between the groups was that the number of days in MHOP was approximately 3.5 times longer for Samaritan participants than those who opted out of Samaritan. The average income from contributions by donors on the platform over three months for those who opted in was $48.80 (SD = 53.75).

The Wilcoxon rank sum test, a non-parametric test, was utilized for all inferential statistics because the population sample was non-normal. The variable *Days in Program *was statistically significant (Z = -3.654, p < 0.001). Though not statistically significant, there was a trend toward an overall decrease in SDOH needs for Samaritan participants (average total Health Leads Social Need Screening Tool score of 5) compared to the non-Samaritan counterparts (score of 6) with an average total Health Leads Social Need Screening Tool score of 5 among Samaritan participants and 6 among non-participants. Among the individual Health Leads Social Needs Screening Tool questions, *Housing* (“Are you worried that in the next 2 months, you may not have stable housing?”) was statistically significant (Z = -2.002, p = 0.045). Of note, none of the participants reported having young children and therefore did not require childcare services. Table 1 displays the study results.

**Table 1:** Inferential statistics.

	Opt-in	Opt-out	Wilcoxon W	Standardized test statistic	P-value
Days in program	435.40 (290.563)	122.67 (187.464)	166.000	-3.654	<0.001
Age	42.47 (13.323)	41.50 (15.036)	343.000	-0.320	0.749
Social determinants of health			457.000	1.888	0.059
Food			1,391.500	-0.587	0.557
Utilities			1,379.500	-0.884	0.377
Housing			1,319.000	-2.002	0.045
Child care			360.000	0.000	1.000
Healthcare			1,400.000	-0.236	0.813
Transportation			348.500	-0.569	0.569
Literacy			1,355.500	-1.393	0.164
Companionship			1,404.000	-0.505	0.613

## Discussion

The purpose of this study was to examine whether MHOP participants reported fewer SDOH needs by accessing Samaritan, an online digital support platform. Our findings revealed that the longer a patient was in the MHOP, there was a trend toward decreased SDOH needs, in particular, housing needs that were significantly different. This trend suggests that the length of time in MHOP may have a considerable impact on an individual’s opportunity to improve his or her needs.

This study also suggests access to a digital technology such as Samaritan might help improve SDOH needs for disadvantaged mental health patients. The interrelationship between program accessibility via the Samaritan app, duration of active MHOP involvement, and decreased SDOH needs demonstrates a potential advantage of digital technology use in improving patient outcomes and social circumstances. For example, technology can facilitate the integration of multiple data sources to assess SDOH for new insights [7]. With the use of advanced analytic methods, including the use of artificial intelligence-based algorithms, technology may enhance a provider’s ability to provide personalized care and comprehensive, effective care management [8,9].

Digital apps often lack attention to SDOH such as lower-income households, low literacy rates, and residence in disadvantaged communities [10] which leads to disparities in care [11]. However, the advantage and distinguishing feature of the Samaritan app compared to other digital resources is that, in addition to identifying and assessing SDOH needs, the app provides a way to mediate interventions needed to improve an individual’s health and social needs through the involvement of community stakeholders. This app provides a novel solution to not only assessing needs but also addressing how community intervention can also help to improve these needs, especially given the limited research on initiatives of similar nature.

A limitation of the present research was both the small number of those who participated in the overall study, and the even smaller number who declined to opt in compared to those who did. Therefore, this study has low generalizability given the small sample size and gender distribution with a majority of male participants. However, despite this statistic, it should be noted that, in general, this statistic is representative of the demographics of the incarcerated population, given that 93% of the Florida inmate population is male [12].

Another limitation is that although the use of the health screening tool helps in the assessment of individual SDOH needs, it is limited in its ability to accurately assess those with extreme SDOH deficiencies. For instance, one question on the screener asks about the threat of utility service shutdown. For individuals who identify as homeless, this question may be difficult to answer accurately, as both answers imply access to housing.

Future studies should investigate alternative measures of SDOH that better capture the needs of the chronically, severely mentally ill who find themselves involved with the criminal justice system. Additionally, the exploration of a minimum amount of time to participate in MHOP before approaching a patient to consent to a digital support app may provide valuable information in the future. Finally, future studies can look more at assessing how the incorporation of reinforcement apps like Samaritan influences an individual’s self-efficacy as self-efficacy is an important component in driving health behaviors [13].

## Conclusions

Among the individual Health Leads Social Needs Screening Tool questions, *Housing* was statistically significant, suggesting access to a digital technology such as Samaritan might help improve SDOH housing needs. However, there was also a significant difference between the groups in the number of days enrolled in MHOP, with those who opted into Samaritan being enrolled approximately 3.5 times longer than those who opted out. Therefore, access to digital technologies, such as Samaritan, might help offenders with mental illness adjust to the many challenges they face upon reentry into the community. As such, these devices may represent one means for improving SDOH needs for disadvantaged mental health patients.
